# Shigella Bacteremia in an Immunocompetent Patient

**DOI:** 10.7759/cureus.19778

**Published:** 2021-11-20

**Authors:** Hayden E Rotramel, Harris S Zamir

**Affiliations:** 1 Internal Medicine, University of Missouri-Kansas City School of Medicine, Kansas City, USA

**Keywords:** antibiotic resistance, type 2 diabetes mellitus, hiv/aids, bacteremia, shigella

## Abstract

Isolation of *Shigella* in the bloodstream is a rare sequela of *Shigella* infections. Shigellemia typically occurs in patients with immature immune responses or in immunocompromised adults. Herein, we present a case of shigellemia in a 40-year-old male who presented with diabetic ketoacidosis (DKA), severe diarrhea, hypovolemic hyponatremia, and altered mental status. Stool cultures were found to be positive for *Shigella*, and broad-spectrum antibiotic therapy was initiated. Because of the patient’s reported sexual exposures, a rapid HIV point of care test was done and returned negative. In spite of intervention, the patient’s vitals, labs, and symptoms failed to improve, and he developed septic shock requiring pressor support in the intensive care unit. Further workup for the etiology of the patient’s sepsis included a CT abdomen and pelvis which showed findings concerning infectious colitis. Blood cultures later returned positive for *Shigella*, which was found to be resistant to multiple antibiotics. The patient was started on IV ceftriaxone with an improvement of and eventual resolution of symptoms. Shigellemia is a rare complication of infection with *Shigella* and necessitates further workup to avoid overlooking potential predisposing factors such as HIV or other immunocompromising conditions. Its susceptibilities should also be evaluated, as *Shigella* strains are more frequently becoming resistant to antibiotics that had previously been the therapies of choice.

## Introduction

The *Shigella* species are Gram-negative, non-spore-forming, facultative anaerobic bacilli that have the potential to cause diarrheal disease by invading colonic epithelium. The global incidence of shigellosis is estimated to be approximately 165 million episodes per year [1]. Immunocompromised adults and children under the age of 5 years are most commonly impacted by *Shigella* infections [2]. *Shigella* infection is most commonly transmitted through the ingestion of contaminated food and water, disproportionately affecting those individuals living in poor and crowded communities without access to adequate sanitation or clean water. However, it can also be passed during oral-anal sexual activity, which puts men who have sex with men (MSM) at risk, especially in those that are immunocompromised [3]. Shigellosis rarely affects immunocompetent individuals.

We report a case of *Shigella flexneri *bacteremia that occurred in a 40-year-old caucasian homosexual man determined to be HIV-negative.

## Case presentation

A 40-year-old Caucasian man with a past medical history of uncontrolled type 2 diabetes mellitus with peripheral neuropathy, alcohol abuse, and chronic pancreatitis presented to the emergency department for a three-day history of profuse watery diarrhea, shortness of breath, and ataxia. 

Diarrhea reportedly began soon after the patient had attended a group barbecue. Initially, the patient experienced two to three loose stools per day, which gradually occurred more and more frequently, up to one to two times per hour at the time of initial presentation. In addition to profuse diarrhea, the patient also became progressively short of breath and confused. The patient denied knowledge of any other guests experiencing similar symptoms, including his partner. He also denied any other sick contacts or consumption of undercooked food. He denied any melena or hematochezia. The patient’s vital signs were remarkable for tachypnea and tachycardia. On physical examination, he was in mild acute distress, covered in feces, with very dry mucous membranes and obvious tachypnea. He was initially alert and oriented to person, place, time, and situation.

In the ED, initial labs were notable for sodium of 114, creatinine of 1.44 (likely secondary to severe diarrhea and resultant dehydration), glucose of 379, elevated anion gap at 32, and arterial blood gas (ABG) showing pH of 7.006, PCO2 of 9, HCO3 of 2, and urinalysis positive for ketones, consistent with acute diabetic ketoacidosis (DKA). 

A *Clostridium difficile* antigen test was performed and returned negative. Stool studies including stool Shiga toxin, *Campylobacter* antigen, and stool culture, as well as blood cultures were obtained. Due to the patient’s reported sexual exposures, a rapid HIV test was also done to assess the patient’s immune status, which returned negative. The patient was then admitted to the intensive care unit (ICU) for further management of acute DKA and hypovolemic hyponatremia secondary to severe diarrhea with unclear etiology.

**Figure 1:**
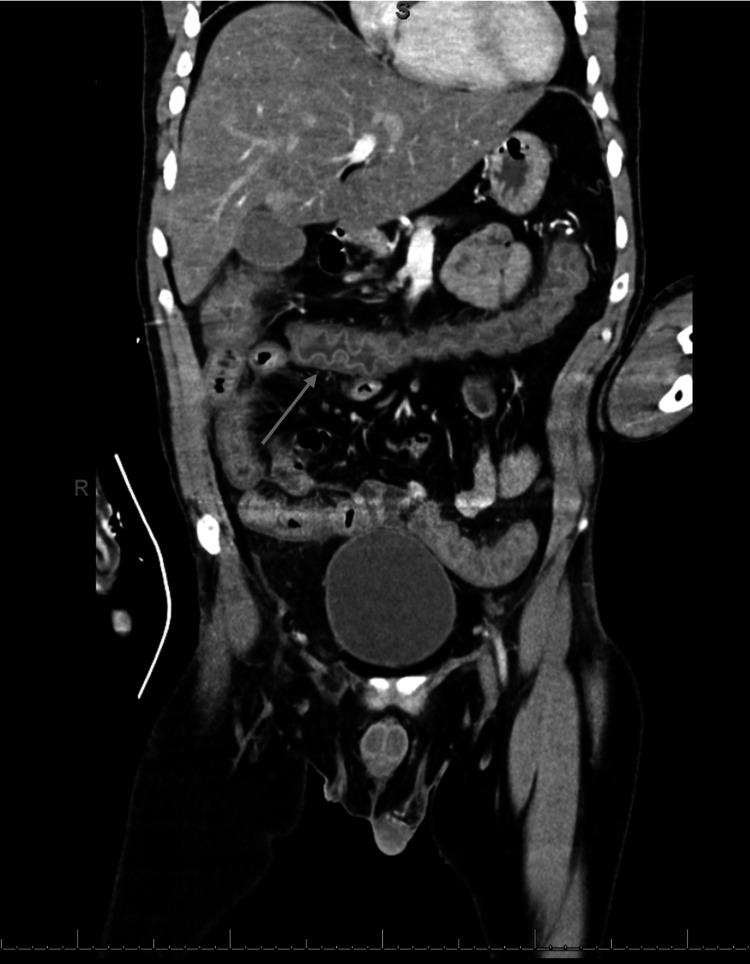
CT abdomen and pelvis with contrast, coronal plane showing diffuse colonic wall thickening

**Figure 2:**
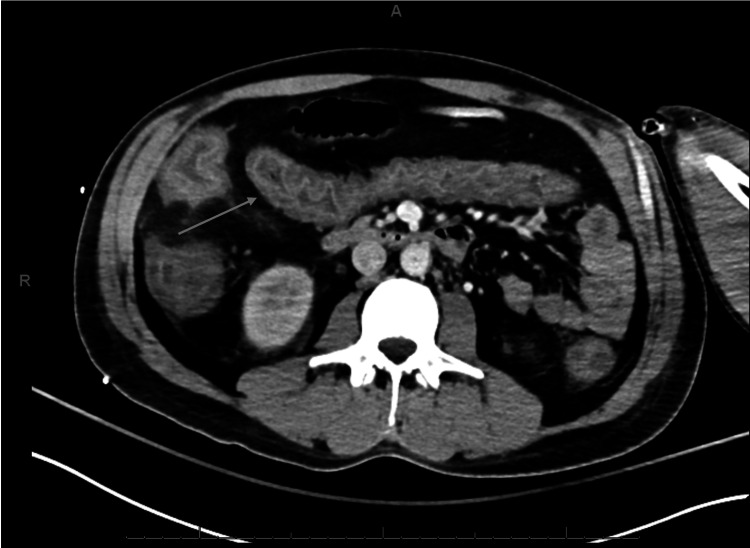
CT abdomen and pelvis with contrast, axial plane showing diffuse colonic wall thickening

Over the next 24 hours, the patient’s anion gap closed and DKA resolved, however, he became febrile, hypotensive (systolic blood pressures in the low 80’s and diastolic blood pressure in the low 50’s), and his mental status became increasingly altered (alert and oriented only to person) raising concern for septic shock. CT abdomen and pelvis with contrast was obtained to evaluate for a source of infection and was notable for diffuse colonic wall thickening which was concerning for infectious colitis (Figures 1, 2).

The patient was started on broad-spectrum antibiotic therapy with vancomycin and Zosyn, without any decrease in stool output (max output within a 24-hour period was approximately 5200mL), resolution of fever, or improvement in mental status. Stool and blood culture results returned positive for *Shigella flexneri *on hospital day three and showed resistance to ampicillin, ampicillin/sulbactam, and trimethoprim/sulfamethoxazole. At this point, infectious disease was consulted for treatment recommendations, and the patient was started on ceftriaxone 2g IV every 24 hours to be continued for two weeks once negative blood cultures were obtained. Due to the unclear route of transmission (food-borne vs anal-oral), it was also recommended that a repeat HIV RNA test be performed three weeks after the initial test was completed to confirm HIV status. After ceftriaxone therapy was initiated, the patient’s vitals improved and stabilized, daily stool output steadily trended downward, and his mental status gradually returned to baseline. 

The patient had a peripherally inserted central catheter (PICC)-line placed to allow him to be discharged home to complete the course of IV ceftriaxone. Prior to discharge he was advised to avoid sexual intercourse for at least one week after the cessation of diarrhea and was counseled on following safe sexual practices for several weeks after, as *Shigella* can remain in the stool for an extended period of time.

## Discussion

The harsh environment of the human digestive tract typically limits *Shigella* infections to generalized colonic inflammation that can be managed with oral rehydration and antibiotics [4]. In rare cases, *Shigella* causes bacteremia which doubles the mortality rate from the infection [5].

Shigellemia is exceedingly rare. A study conducted by Tobin-D’Angelo et al. in the Georgia Department of Public Health reviewed data from 2002 to 2012 and found that of 11,262 cases of *Shigella* infection, only 72 (0.64%) patients were found to have positive blood cultures. The study found that patients who developed shigellemia were most commonly >18 years of age, black in race, and 51% were HIV infected [6].

Our patient was neither black nor HIV positive and thus had an unusual presentation of shigellemia. Perhaps, our patient’s uncontrolled type 2 diabetes mellitus may have been a risk factor in his development of this infection. Chronic hyperglycemia causes a dysfunctional immune response and thus immune compromise [7]. However, a more thorough understanding of why this immune dysfunction occurs is needed to improve the outcomes of infectious disease treatment and prevention in diabetics.

Treatment of *Shigella* infections is further complicated by the emergence of antimicrobial resistance. The World Health Organization currently recommends the use of fluoroquinolones as the therapy of choice for shigellosis, but due to the emergence of ciprofloxacin resistance in *Shigella*, therapeutic management has recently shifted to the use of cephalosporins instead, which were successfully used in treating our patient [8,9]. However, strains of *Shigella flexneri* continue to demonstrate increasing resistance to cephalosporins, azithromycin, and fluoroquinolones. Thus, care should be taken to review sensitivities and treat patients appropriately. [10].

After treatment of the acute illness, patients should be counseled on prevention techniques. This includes obvious interventions such as handwashing, but the CDC also recommends avoidance of sex by men who have sex with men until resolution of diarrhea [11].

## Conclusions

In patients presenting with shigellemia, it is imperative to evaluate all risk factors. This is particularly true for patients who otherwise have no identifiable cause for an immunocompromised state. In shigellemia or disseminated shigellosis, it is also important to test for antibiotic susceptibilities due to rising antibiotic resistance. The mainstay of prevention is to counsel patients on safe practices after the infection resolves in order to avoid further spread to close contacts.
